# Longitudinal volumetric changes in amygdala subregions in frontotemporal dementia

**DOI:** 10.1007/s00415-023-12172-5

**Published:** 2024-01-24

**Authors:** Mengjie Huang, Ramon Landin-Romero, Sophie Matis, Marshall A. Dalton, Olivier Piguet

**Affiliations:** 1 School of Psychology, The University of Sydney, Camperdown, NSW 2050 Australia; 2 Brain and Mind Centre, The University of Sydney, 94 Mallett Street, Camperdown, NSW 2050 Australia; 3 School of Health Sciences, The University of Sydney, Camperdown, NSW 2050 Australia

**Keywords:** Amygdala, Structural MRI, FreeSurfer, Frontotemporal dementia, Alzheimer’s disease

## Abstract

Amygdala atrophy has been found in frontotemporal dementia (FTD), yet the specific changes of its subregions across different FTD phenotypes remain unclear. The aim of this study was to investigate the volumetric alterations of the amygdala subregions in FTD phenotypes and how they evolve with disease progression. Patients clinically diagnosed with behavioral variant FTD (bvFTD) (*n* = 20), semantic dementia (SD) (*n* = 20), primary nonfluent aphasia (PNFA) (*n* = 20), Alzheimer’s disease (AD) (*n* = 20), and 20 matched healthy controls underwent whole brain structural MRI. The patient groups were followed up annually for up to 3.5 years. Amygdala nuclei were segmented using FreeSurfer, corrected by total intracranial volumes, and grouped into the basolateral, superficial, and centromedial subregions. Linear mixed effects models were applied to identify changes in amygdala subregional volumes over time. At baseline, bvFTD, SD, and AD displayed global amygdala volume reduction, whereas amygdala volume appeared to be preserved in PNFA. Asymmetrical amygdala atrophy (left > right) was most pronounced in SD. Longitudinally, SD and PNFA showed greater rates of annual decline in the right basolateral and superficial subregions compared to bvFTD and AD. The findings provide comprehensive insights into the differential impact of FTD pathology on amygdala subregions, revealing distinct atrophy patterns that evolve over disease progression. The characterization of amygdala subregional involvement in FTD and their potential role as biomarkers carry substantial clinical implications.

**Supplementary Information:**

The online version contains supplementary material available at 10.1007/s00415-023-12172-5.

## Background

Frontotemporal dementia (FTD) refers to a group of progressive neurodegenerative disorders associated with predominant frontal and/or temporal lobe atrophy [1]. FTD is clinically heterogenous and encompasses three main phenotypes: a behavioal variant (bvFTD), semantic dementia (SD), and progressive nonfluent aphasia (PNFA). BvFTD is characterized by profound behavior and personality changes [2, 3], with atrophy predominantly observed in the frontal and anterior temporal lobes, particularly affecting the insula, orbitofrontal, and medial prefrontal cortices [4]. SD is marked by progressive non-modality specific loss of semantic knowledge [5]. Brain imaging in SD consistently report marked asymmetric (usually more left lateralized) anterior temporal lobe atrophy [6, 7]. PNFA typically presents with impaired expressive speech and language skills [2, 5] and atrophy in the left inferior frontal gyrus and anterior insula [8, 9].

In addition to cortical damage, FTD also involves changes in subcortical brain regions such as the amygdala. The amygdala is located in the medial temporal lobes and plays a central role in various cognitive and socio-emotional processes such as memory and learning [10]. A plethora of cross-sectional studies have found significant amygdala damage in all clinical forms of FTD [6, 11–16]. Yet, they have yielded inconsistent results, and only a few have directly compared amygdala changes across FTD phenotypes. For instance, while some studies have found bilateral and symmetrical amygdala atrophy in bvFTD [13, 17, 18], others have not [12, 19, 20]. In contrast, pronounced and asymmetrical amygdala atrophy (usually left greater than right) has been reported in SD, with variable involvement of the right amygdala [6, 19, 21]. Furthermore, previous findings on amygdala atrophy in PNFA are limited and inconclusive, with studies reporting bilateral [11] or left-confined [19] atrophy compared to controls. These discrepancies may reflect variances in methodology (e.g., whole brain versus a-priori region of interest approach; manual versus automated segmentation), patient characteristics (e.g., disease duration and severity), and sample sizes.

The few existing longitudinal investigations of amygdala volumes in FTD have also yielded mixed results. A recent case–control study of bvFTD patients found no progressive atrophy over 12–16-months [17], contrasting to Bejanin [13], which detected greater volume loss in bvFTD compared to controls over a similar follow-up duration. Of note, several studies have used atlases that combined amygdala with the hippocampus [22–25], making it difficult to determine to what extent each structure contributed to the observed decline. Research that directly compare progression trajectories of amygdala atrophy among FTD phenotypes is also notably lacking.

Crucially, the amygdala is a heterogenous structure comprising several nuclei with widespread interconnections with other cortical and subcortical brain regions [26, 27]. These nuclei are traditionally grouped into three subregions based on their anatomical and functional characteristics [26–28]. The basolateral (BLA) subregion receives substantial inputs from sensory cortices and is reciprocally connected with the frontal cortex, thalamus, hippocampus, and the occipital lobes [26, 29, 30]. Functionally, the BLA subregion has been shown to be involved in decision-making, memory, and fear learning [31]. The superficial (SUP) subregion plays a key role in processing olfactory and social information [26], while the centromedial (CeM) subregion serves as the main output region that mediates motor behaviors such as flight reaction and startle responses, via its projections to the hypothalamus, basal forebrain, and the brainstem regions [26, 32]. Advances in imaging techniques have made it possible to differentiate these nuclei on standard structural magnetic resonance imaging (MRI) in healthy and clinical populations in vivo [33]. Recent research has identified specific amygdala nuclei volume reductions in the genetic forms of FTD [34], the extent of such changes in the clinical forms of FTD, however, has not yet been explored.

Overall, previous research on amygdala atrophy in FTD has shown inconsistencies, with few studies directly comparing amygdala volumes across all FTD phenotypes. In addition, no previous research has examined whether amygdala subregions undergo distinct volumetric trajectories with disease progression. To address these gaps, this study aimed to investigate the profiles of amygdala atrophy in each clinical form of FTD, and track these changes at the subregional level over the disease course. Here, we applied a cutting-edge automated segmentation technique and accounted for intra-individual variability over time within a longitudinal cohort of well-characterized clinically diagnosed FTD patients, to establish the rates of atrophy of amygdala subregions in the main FTD syndromes. Considering the existing evidence, we hypothesized that all FTD variants would exhibit reduced amygdala volumes relative to healthy controls at baseline, with SD showing more severe amygdala atrophy compared to the other groups. We did not formulate any specific hypotheses of longitudinal trajectories of subregional change due to the scarcity of existing literature in this regard. Importantly, we also included typical Alzheimer’s disease (AD) as a disease control group of interest. Amygdala atrophy is frequently reported in AD, and some previous studies have shown comparable extent of amygdala atrophy and rates of annual progression between AD and bvFTD [20, 24, 35]. Our study also aimed to uncover any potential differences in specific amygdala subregions between these diseases.

## Methods

### Participants

Eighty patients with a clinical diagnosis of FTD or AD (twenty bvFTD, twenty SD, twenty PNFA, twenty AD) were selected from the FRONTIER Research Clinic database (Sydney, Australia). Diagnosis was made in accordance with current clinical diagnostic criteria for probable FTD or AD [2, 5, 36], based on neurological, neuropsychological, and neuroimaging examination, as well as informant report. Twenty healthy adults who matched to patients for age, sex, and education were also included in the study. All patients completed clinical, cognitive, and neuroimaging assessments at least on two occasions at annual intervals.

For all participants, exclusion criteria included concurrent neurological or psychiatric diagnosis, history of substance or alcohol abuse, or limited English skills. SD patients with predominant right temporal lobe atrophy at presentation were also excluded from the study to ensure a homogeneous sample. Informed consent was obtained from all participants prior to assessment in accordance with the Declaration of Helsinki. The study was approved by the South Eastern Sydney Local Health District and the ethics committees of the University of New South Wales and the University of Sydney.

### Measures of cognitive function and disease severity

Global cognitive functioning was assessed by the Addenbrooke’s Cognitive Examination-Revised (ACE-R) [37] or ACE-III [38]. The ACE, which measures the integrity of five cognitive domains: memory, attention, language, fluency, and visuospatial skills, is scored out of 100 points with higher scores indicating better cognitive abilities. A score below 88 suggests the presence of a cognitive impairment [38, 39]. Before analyses, where applicable, ACE-R scores were converted to ACE-III scores using the conversion formula as previously described [39].

The Frontotemporal dementia Rating Scale (FRS) was used to determine disease severity and functional impairment of patients [40]. The FRS is a 30-item questionnaire rated by carers. Total raw score was first converted to a percentage to account for premorbid abilities, and then converted again to a logit score that ranges from 5.39 to—6.66. Higher scores indicate better functioning.

### MRI acquisition

All participants underwent a whole-brain 3D structural MRI scan within 6 months of their clinical assessment at baseline and follow-up visits. A total of 250 T1-weighted scans were acquired using two equivalent 3 T scanners. Most MRIs (*n* = 205; 82%) were acquired on a Philips 3 T scanner, while the remaining (*n* = 45; 18%) were acquired on a GE Discovery MR750 scanner. To ensure the comparability of the T1-weighted images, all scans were obtained using a standard eight-channel head coil and harmonized protocols: 256 × 256 matrix, 200 slices, slice thickness 1 mm, 1 × 1 mm in-plane resolution, echo time/repetition time = 2.6/5.8 ms, flip angle *α* = 8.

### MRI data processing

#### Cross-sectional data processing

Baseline T1-weighted images were pre-processed using the FreeSurfer V.7.1.1 mainstream pipeline ("recon-all" library tool) for whole brain segmentation (http://surfer.nmr.mgh.harvard.edu). In brief, the images were first skull-stripped and removed of non-brain tissues. The brain-extracted images were affinely registered with Montreal Neurological Institute (MNI305) atlas space using a FreeSurfer script known as Talairach, and then segmented into subcortical white matter and deep grey matter [41]. All resulting images were visually inspected for segmentation accuracy and passed quality check before proceeding with amygdala nuclei segmentation.

**Fig. 1:**
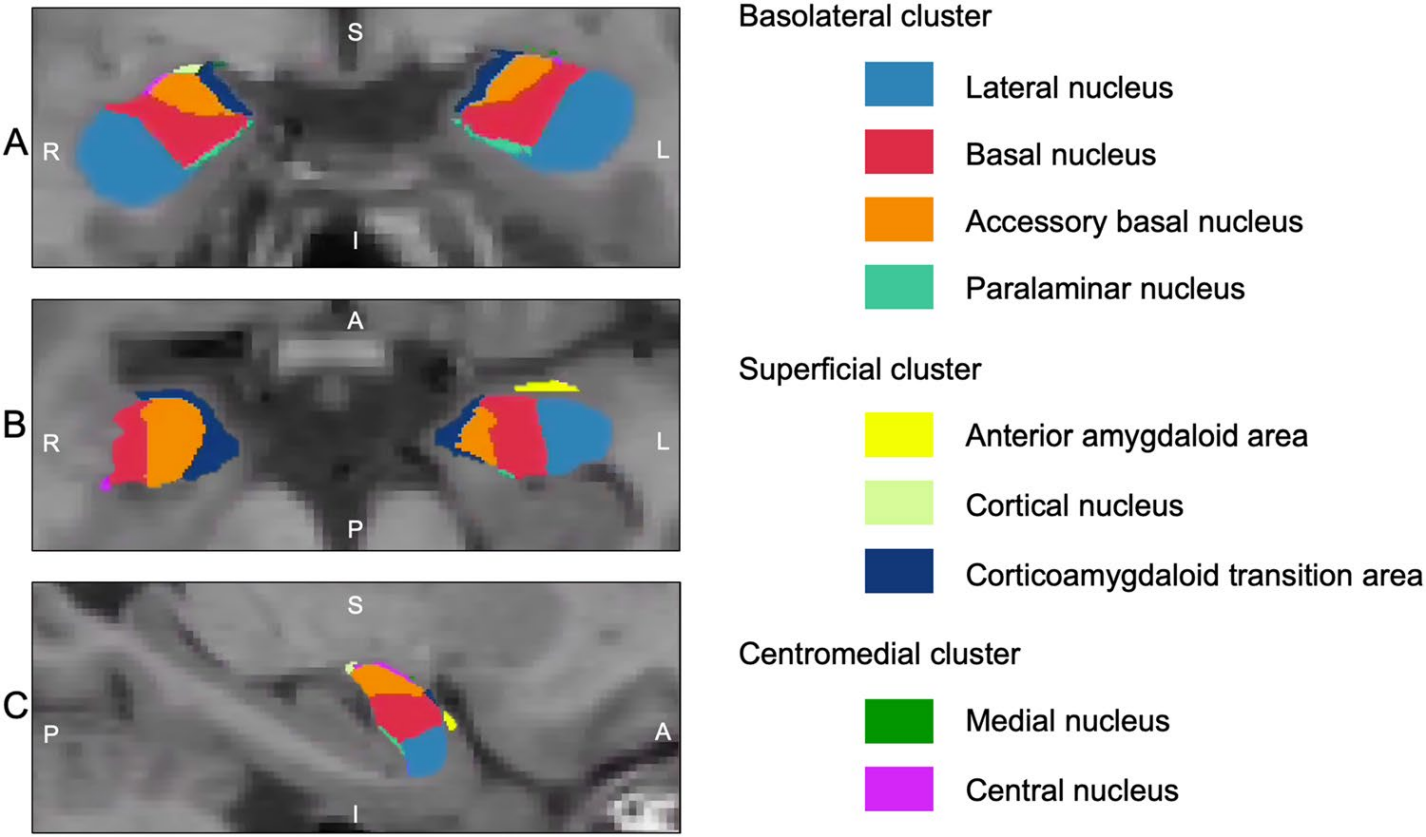
Visualization of amygdala nuclei in a healthy control subject using FreeSurfer V.7.1.1 amygdala nuclei segmentation pipeline. **A**–**C** represent the coronal, axial, and sagittal views. *R* right, *L* left, *S* superior, *I* interior, *A* anterior, *P* posterior

Amygdala nuclei were segmented using the FreeSurfer V.7.1.1 cross-sectional segmentation pipeline. This protocol performs a joint segmentation of hippocampus subfields and amygdala nuclei to prevent overlapping or gaps between these adjacent structures. The amygdala was automatically segmented into nine labeled nuclei (i.e., basal nucleus, lateral nucleus, accessory basal nucleus, paralaminar nucleus, cortical nucleus, central nucleus, medial nucleus, cortico-amygdaloid transition area, and anterior amygdaloid area) for left and right hemisphere based on in vivo boundary contrasts [33] (Fig. 1).

Finally, amygdala nuclei volumes for each participant were exported from FreeSurfer in mm^3^, and then converted to a ratio of the total intracranial volume (TIV) to correct for different head sizes using the formula: amygdala volume/TIV *1000. Individual nuclei volumes were then summed into three subregions in line with previous literature: the BLA subregion (involving the lateral, basal, accessory basal, and paralaminar nuclei), the SUP subregion (involving the cortical nucleus, cortico-amygdaloid transition area, and anterior amygdaloid area), and the CeM subregion (involving the central and medial nuclei) (Fig. 1). These subregions were extracted separately for each hemisphere.

#### Longitudinal data processing

Longitudinal data were processed using the FreeSurfer V.7.1.1 longitudinal pipeline [42]. Healthy controls were excluded from these analyses as it was assumed that they would not exhibit significant changes in amygdala volumes over time. First, all 230 T1-weighted images from the patient groups were cross-sectionally pre-processed using the default FreeSurfer workflow (“recon-all”) for whole brain segmentation. Next, an unbiased within-subject template was generated for each individual from all available time points using inverse consistent registration [43]. Following template creation, subsequent processing steps such as skull stripping, Talairach transforms, atlas registration, cortical surface construction, and subcortical parcellation were initiated with common information from the within-subject template, thereby enhancing both reliability and statistical power [42].

After the data were processed with the longitudinal stream, amygdala nuclei were segmented using the longitudinal version of the FreeSurfer V.7.1.1 segmentation pipeline. This protocol uses unbiased individual templates to refine the segmentation of amygdala nuclei across different time points, enabling a more accurate capture of changes in amygdala nuclei over time [44]. Finally, the nuclei volumes for each individual were exported from FreeSurfer, corrected by TIV and summed into the three relevant subregions as previously described, in preparation for the subsequent group-level analysis using the linear mixed effects (LME) models.

### Statistical analyses

All statistical analyses were performed using SPSS V.27.0 (IBM SPSS Statistics) and figures were created using GraphPad Prism 9 (GraphPad Software, San Diego, CA, USA). *p* values less than 0.05 are considered statistically significant.

#### Cross-sectional analyses

Cross-sectional analyses were conducted to compare demographic characteristics and amygdala subregional volumes across groups at baseline visit. An amygdala asymmetry index was calculated for each individual to assess laterality using the formula: (right hemisphere amygdala volume—left hemisphere amygdala volume)/total amygdala volume *100. Positive index values indicate greater left than right atrophy, whereas negative index values indicate greater right than left atrophy. Continuous variables were analyzed using one-way analysis of variance (ANOVA) followed by Sidak post hoc tests to adjust for multiple comparisons. Categorical variables such as sex were compared using Chi-squared tests (X^2^). Covariates were not included as potential confounders such as age, sex, and disease duration were matched across the groups (Table 1).

**Table 1:** Demographic and clinical characteristics of study participants at baseline

	bvFTD *n* = 20	SD *n* = 20	PNFA *n* = 20	AD *n* = 20	HC *n* = 20	*F*	*p* value	Post hoc test (Sidak)
Age (Y)	63.2 ± 7.7	63.4 ± 6.7	64.4 ± 10.5	62.8 ± 6.9	68.2 ± 5.2	1.705	ns	-
Sex (M:F)	12:8	12:8	9:11	10:10	9:11	1.843^a^	ns	-
Education (Y)	11.4 ± 2.5	12.3 ± 3.2	12.8 ± 2.7	12.7 ± 3.2	13.4 ± 2.5	1.365	ns	-
Disease duration (Y)	3.7 ± 1.9	4.1 ± 1.5	3.4 ± 2.0	3.8 ± 1.3	-	0.580	ns	-
ACE-III, total score (max: 100)	76.6 ± 10.4	62.5 ± 16.2	81.2 ± 9.6	72.2 ± 11.1	94.6 ± 2.8	17.406	< .001	SD < bvFTD*, PNFA**, HC**; bvFTD, PNFA, AD < HC**
FRS Rasch total score	0.2 ± 1.09	1.9 ± 1.52	2.7 ± 1.07	1.2 ± 1.70	–	11.185	< .001	bvFTD < SD*, PNFA**; AD < PNFA*

Values are means ± standard deviations; missing data: ACE-III total scores: 4bvFTD, 3SD, 3PNFA, 3SD, 3AD, 6HC; FRS Rasch total score: 1bvFTD, 1 PNFA

*bvFTD* behavioral variant frontotemporal dementia, *SD* semantic dementia, *PNFA* progressive nonfluent aphasia, *AD* Alzheimer’s disease, *HC* healthy controls, *ACE-III* Addenbrooke’s Cognitive Examination-Third edition, *FRS* Frontotemporal dementia Rating Scale

^a^Chi-square test

^*^p < .05; **p < .001

#### Longitudinal analyses

For longitudinal analyses, the LME models were used to examine changes in TIV-corrected amygdala subregional volumes in all groups over time. The LME models provide a powerful framework for analyzing longitudinal data by incorporating all available data, addressing issues such as single time observations and variable missing rates or timing of observations [45]. This flexibility provides insights into the complex dynamics of temporal trajectories of progression in the amygdala subregions. In the present study, the fixed effects of the model included diagnosis, follow-up time in years (from the first MRI scan), and the interaction between diagnosis and follow-up time. The random effect of the model included individual variability associated with a patient at baseline (using random intercept model). To summarize the rates of amygdala volume loss, longitudinal atrophy rates were expressed as annual percentage change using the formula: (TIV-corrected most recent volume − TIV-corrected baseline volume)/TIV-corrected baseline volume/time intervals *100.

## Results

### Demographics

Participants’ baseline and clinical characteristics are reported in Table 1. Groups were matched for age, sex, and years of education. The clinical groups were also matched for disease duration at baseline visit. Significant overall group differences were found in functional ability (FRS Rasch score; *p* < 0.001) and cognitive functioning (ACE-III total score; *p* < 0.001). On the FRS, bvFTD patients showed significantly greater impairment compared to SD (*p* = 0.003) and PNFA patients (*p* < 0.001). On the ACE-III, all clinical groups performed significantly worse than healthy controls (all *p* values < 0.05). Not surprisingly, given the language load of the task, SD demonstrated the greatest cognitive impairment relative to all other patient groups. No other differences across the clinical groups were found on the ACE-III (Table 1). Number of scans per group and average time from baseline at each time point are reported in Table 2.

**Table 2:** Number of scans per group and average time from baseline at each time point

	bvFTD	SD	PNFA	AD	HC	Time from baseline (Y)
Number of scans						
Baseline	20	20	20	20	20	0
Year 1	17	19	19	20	–	1.03 ± 0.15
Year 2	11	12	9	12	–	2.06 ± 0.17
Year 3	9	6	7	9	–	3.01 ± 0.24
Total	57	57	55	61	20	–

Values are means ± standard deviations

*bvFTD* behavioral variant frontotemporal dementia, *SD* semantic dementia, *PNFA* progressive nonfluent aphasia, *AD* Alzheimer’s disease, *HC* healthy controls

### Baseline amygdala volume results

#### Total volumes

Compared to controls, bvFTD, SD, and AD displayed significant amygdala volume reduction (Supplementary Table 1). In contrast, the amygdala appeared to be relatively intact in PNFA, which showed comparable volumes with the controls. Within the clinical groups, the greatest volume reduction was observed in SD, in which the left amygdala volume was significantly smaller than in all the other groups (all *p* values < 0.001). The right amygdala in SD also showed greater atrophy compared to PNFA (*p* < 0.001). In addition, bvFTD and AD both showed smaller amygdala volumes than PNFA (both *p* values < 0.05), although the difference did not reach significance for the left amygdala between AD and PNFA (*p* = 0.123).

Further, a one-way ANOVA was carried out to investigate the magnitude of amygdala atrophy asymmetry across groups. This analysis revealed significant group differences in amygdala asymmetry index (*p* < 0.001). Post hoc tests showed that the SD group exhibited a significant larger asymmetry index than all the other groups (all *p* values < 0.001), with a greater left to right amygdala atrophy. In contrast, the asymmetry index did not differ among bvFTD, PNFA, AD, and controls, indicating comparable extent of amygdala asymmetry among these four groups.

#### Subregional volumes

**Fig. 2:**
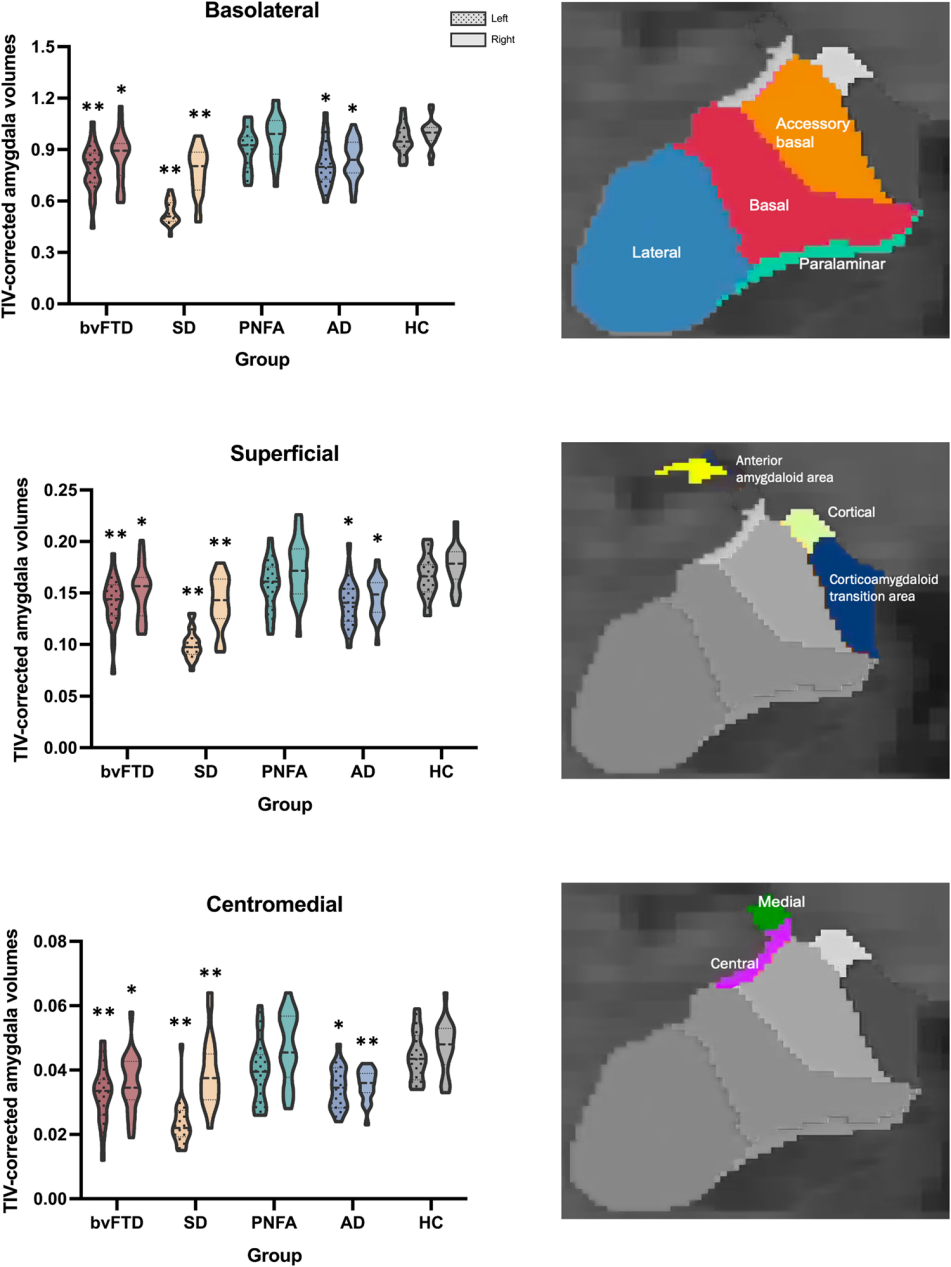
Violin plots of corrected amygdala total and cluster volumes at baseline. Asterisk indicates significant difference compared to controls at *p < .05, **p < .001. *TIV* total intracranial volume, *bvFTD* behavioral variant frontotemporal dementia, *SD* semantic dementia, *PNFA* progressive nonfluent aphasia, *AD* Alzheimer’s disease; *HC* healthy controls

Consistent with total volume findings, subregional volume analyses revealed widespread significant atrophy in all subregions in bvFTD, SD, and AD relative to the controls (Fig. 2; Supplementary Table 1). No significant differences were observed between PNFA and controls for any of the subregions (all *p* values > 0.05).

Within the clinical groups, SD showed the smallest volumes for all three subregions on the left side compared to the other groups (*p* < 0.001 for all comparisons). For the right side, SD showed significantly smaller BLA and SUP volumes than PNFA (all *p* values < 0.05). In bvFTD, bilateral BLA and right CeM subregions displayed greater volume reduction than in PNFA (all *p* values < 0.05). Notably, the AD patients displayed a similar pattern of bilateral volume loss as bvFTD, and no significant differences were found in any subregional volumes between bvFTD and AD.

### Longitudinal amygdala volume results

#### Total volumes

The results from the LME models are provided in Table 3. The models revealed a significant main effect of diagnosis and of time on amygdala volumes bilaterally (all *p* values < 0.001). A significant interaction between diagnosis and follow-up time was observed for the right amygdala (*p* < 0.001), but not for the left (*p* = 0.327), indicating that the decline rates in the right amygdala differed across groups over time. Further examination of this interaction showed that SD exhibited the greatest right amygdala decline compared to all the other groups (all *p* values < 0.001), and PNFA also showed a greater decline rate in the right amygdala than bvFTD (*p* = 0.02). Notably, SD displayed a marked asymmetric pattern of amygdala decline over time, with a greater decline on the right side (− 4.27%/year) compared to the left (− 1.08%/year) (Supplementary Table 2).

**Table 3:** Linear mixed effects models result for longitudinal comparisons of amygdala total and subregional volumes

	Diagnosis		Follow-up time		Diagnosis x follow-up time interaction		Follow-up time* diagnosis post hoc (Sidak)
	F	*p* value	F	*p* value	F	*p* value	
Basolateral							
Left	38.013	< .001	25.652	< .001	0.698	ns	–
Right	10.016	< .001	68.899	< .001	10.290	< .001	SD > bvFTD**, PNFA*, AD**; PNFA > bvFTD*
Superficial							
Left	16.655	< .001	37.512	< .001	1.149	ns	–
Right	8.647	< .001	42.338	< .001	5.459	.001	SD > bvFTD**, AD*; PNFA > bvFTD*
Centromedial							
Left	12.831	< .001	43.869	< .001	1.550	ns	–
Right	7.698	< .001	30.339	< .001	1.334	ns	–
Total							
Left	35.303	< .001	33.914	< .001	1.162	ns	–
Right	10.000	< .001	90.801	< .001	10.168	< .001	SD > bvFTD**, PNFA*, AD**; PNFA > bvFTD*

*bvFTD* behavioral variant frontotemporal dementia, *SD* semantic dementia, *PNFA* progressive nonfluent aphasia, *AD* Alzheimer’s disease, *ns* non-significant (*p* > .05)

^*^p < .05; **p < .001

#### Subregional volumes

**Fig. 3:**
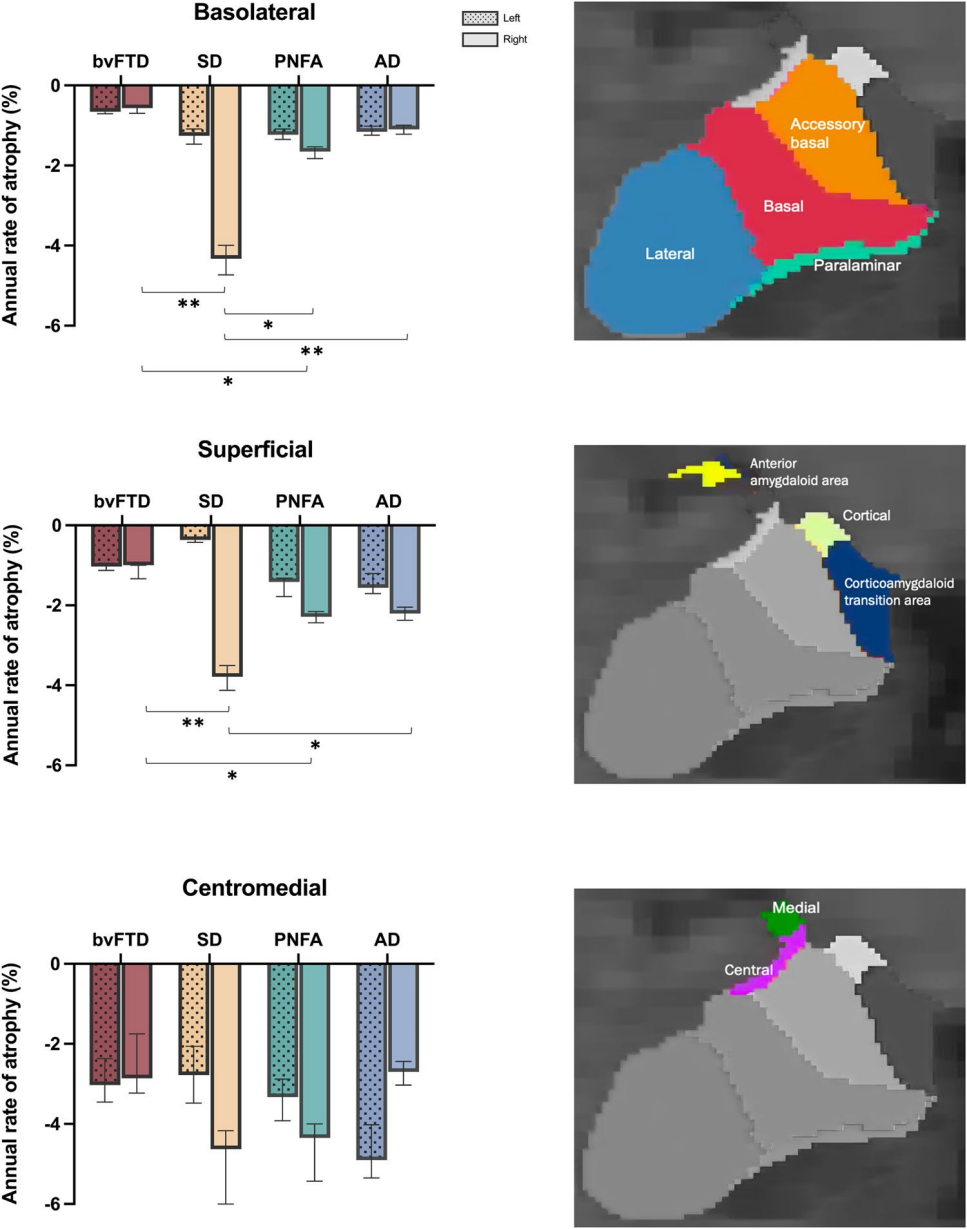
Annual percentage reduction of corrected amygdala total and cluster volumes. SD displayed the greatest rate of annual decline in the right basolateral and superficial clusters compared to all the other groups. PNFA displayed greater rate of annual decline compared to bvFTD in the same subregions. Error bars represent 95% confidence intervals. Asterisk indicates significant difference compared to controls at *p < .05, **p < .001. *TIV* total intracranial volume, *bvFTD* behavioral variant frontotemporal dementia, *SD* semantic dementia, *PNFA* progressive nonfluent aphasia, *AD* Alzheimer’s disease

Consistent with total volume analyses, the LME models revealed a significant main effect of diagnosis and follow-up time on all three subregions (all *p* values < 0.001) (Table 3), suggesting that amygdala subregions experience progressive volume loss as disease progresses. The annual atrophy rates of all subregions are reported in Fig. 3 and Supplementary Table 2.

A significant diagnosis x time interaction was detected only for the right BLA (*p* < 0.001) and right SUP (*p* = 0.001) subregions. Specifically, SD showed the greatest decline in the right BLA (*p* < 0.005 compared to all the other groups) and right SUP subregions (*p* < 0.05 compared to bvFTD and AD). PNFA also showed a greater decline in both subregions compared to bvFTD (both *p* values < 0.05). No significant group differences in decline rates emerged in the CeM subregions.

## Discussion

Applying a novel automated brain segmentation protocol, our study demonstrates distinct amygdala subregional atrophy trajectories with disease progression in the canonical FTD syndromes. In particular, two findings enhance the existing body of knowledge on brain structural changes in FTD. First, our results revealed that the amygdala decline trajectories are driven by volumetric alterations in amygdala subregions specific to each FTD phenotype, with hemisphere-specific patterns. Second, we uncovered that amygdala atrophy presents early in the disease course of bvFTD and SD, whereas it occurs later in the disease course in PNFA. Importantly, the observed atrophy is mediated by changes in different subregions, underscoring the phenotypic heterogeneity across FTD variants. Overall, our systematic investigations provide a thorough understanding of the volumetric changes of amygdala in FTD as disease progresses and highlight differential involvement of amygdala subregions in the clinical forms of FTD. These findings are discussed in detail in the subsequent sections.

Consistent with our hypothesis, overall amygdala volume reduction was most severe in SD at baseline compared with all the other groups. SD also exhibited the most asymmetrical atrophy (left > right), which is a characteristic feature of the left-lateralized SD variant [6]. The degree of asymmetry reduced with disease progression as demonstrated by the disproportionate volume reduction in the right amygdala over time. Our result is in keeping with previous research that documented a greater rate of degeneration in the right temporal lobe in left-lateralized SD patients [46, 47]. We extend this finding by showing that the effect is driven specifically by significant progressive volume loss in the BLA and SUP subregions, and much less so in the CeM subregion. Indeed, the right CeM subregion appeared to be relatively spared over the disease course, whereas the left CeM subregion underwent significant atrophy at baseline but did not show prominent decline over time. Importantly, the BLA and SUP subregions have a similar cytoarchitecture to the cerebral and olfactory cortex. In contrast, the CeM subregion is thought to be an extension of the ventral striatum [48]. These cytoarchitectural differences may potentially contribute to differential vulnerability of the amygdala subregions to progressive degeneration in SD. In addition, the observation that the BLA and SUP subregions are particularly affected in SD is consistent with their known involvement in emotion processing. The functional implications of this differential vulnerability remain to be fully explained.

In bvFTD, we found significant bilateral amygdala atrophy compared to the controls at baseline, albeit less pronounced than that observed in SD, aligning with some previous studies [11, 17, 18]. We further revealed a symmetrical pattern of amygdala damage across all subregions, suggesting a uniformity in the extent of amygdala involvement in bvFTD. Despite an early and severe atrophy at baseline, bvFTD exhibited a less pronounced, and more symmetrical pattern of annual volume decline compared to SD and PNFA, highlighting distinct trajectories of amygdala atrophy progression across FTD phenotypes. It is plausible that more rapid pathological changes in the early phases of the disease are followed by a plateauing phase. Here, our study included scans up to 3.5 years after the baseline assessment, which amounts to half of the average disease duration in bvFTD generally reported. Longer follow-up periods and the use of non-linear modeling approaches will be needed to thoroughly test this hypothesis and provide a comprehensive account of amygdala atrophy trajectory in bvFTD.

In marked contrast to bvFTD and SD, the amygdala was preserved in PNFA at baseline. Over time, however, PNFA demonstrated widespread amygdala volume loss, notably in the right BLA and SUP subregions. Our findings highlight that amygdala volume reduction occurs later in the disease course in PNFA relative to bvFTD and SD. Importantly, while PNFA is described as primarily a language disorder with relative sparing of behavioral abnormalities [5], several studies have reported the emergence of behavioral changes as early as 1-year post-baseline assessment affecting empathy [49]. Others have also reported increased behavioral and emotional disturbances in this group 6 years after baseline assessment [49–51]. Given that behavioral abnormalities are primarily linked with damage to neuroanatomical structures in the right hemisphere, it is plausible that global amygdala volume loss, notably the pronounced decline in the right hemisphere, underlies the emergence of emotional and behavioral disturbances observed in PNFA patients. As aforementioned, the BLA and SUP subregions are associated with roles in coordinating sensory input and processing social information, respectively [26]. Our results point to a possible functional dissociation between the left and right BLA and SUP subregions, with a stronger implication of the right side in social cognition. Whether volume reduction in these specific subregions correlates with the emergence of behavioral symptoms remains to be investigated, which may shed additional light on the functional specificity of amygdala subregions in humans. Moreover, our findings may also partly explain the inconsistent finding of amygdala atrophy in PNFA, as the atrophy may be absent or too subtle to be detected by cross-sectional studies at the time of early presentation.

Notably, we identified a similar profile of amygdala change at baseline and over time in bvFTD and AD. This is in keeping with previous evidence [24, 35], suggesting that alterations in total amygdala volume and/or its subregions do not discriminate between bvFTD and AD. It is well established that patients with bvFTD exhibit a higher prevalence and greater severity of behavioral disturbances compared to patients with AD [52], and these disturbances are often associated with amygdala abnormalities. Some examples include emotion recognition deficits [53, 54], reward deficits [55], and changes in eating behaviors [56, 57]. This seemingly surprising similarity in amygdala atrophy profile between bvFTD and AD may suggest a complex interplay between the amygdala and other brain regions, such as the hippocampus and orbitofrontal cortex, which collectively underpin various cognitive and socio-emotional functions. Several studies using diffusion weighted imaging techniques have reported more prominent and widespread white matter damage in bvFTD compared to AD, especially in the frontal and temporal regions [20, 58]. It is plausible that severe disruption in the connectivity between amygdala and other brain regions in bvFTD underlies their profound behavioral symptoms, despite the similar degree of volume reductions within the amygdala with AD. Yet, to date, little is known about connectivity alterations of the amygdala subregions.

Some potential limitations of the study need to be noted. First, the automated segmentation protocol used in this study employs a probabilistic atlas constructed from high resolution ex vivo MRI data [33]. It has been argued that FreeSurfer may tend to overestimate boundaries when processing low-quality images [59]. We cannot fully exclude that the spatial resolution (1mm^3^) of our T1 MR images may have had an impact on segmentation accuracy. This point, however, was mitigated by our careful manual quality control of all our T1 MR images that were checked for the presence of movement artefacts. Nevertheless, our volume measurements may need to be interpreted with caution, especially in the case of the CeM subregion, which comprises the smallest amygdala subregion and is associated with greater variability. Of relevance here, we quantified annualized volume decline by calculating the percentage of annual change in TIV-corrected amygdala volumes. Given that percentages provide a relative measure of change based on a baseline measurement, small changes in volume can appear significant in percentage terms if the baseline value was small, whereas large changes might appear less impactful when the baseline value was also large. Therefore, when interpreting the longitudinal decline rates, it is important to consider both the size of the subregions and the potential influence of percentage-based measurements on perceived changes. In addition, to minimize the risk of inaccurate boundary placement between nuclei, we clustered individual nuclei into three subregions according to their structural and functional characteristics. As such, changes in specific nuclei within these subregions cannot be discerned. Another possible limitation is our explicit decision to exclude patients with right-lateralized SD, which presents with extensive atrophy in the right temporal lobes, given their small number. Whether the severity and trajectory of amygdala changes in this group mirror those seen in the typical left-lateralized SD will be important to investigate, particularly in light of the renewed interest in this syndrome [60, 61].

In summary, amygdala abnormalities are of clinical relevance in FTD, given its known role in cognitive and socio-emotional functions. Our study confirmed amygdala involvement in all FTD phenotypes, and for the first time, provided longitudinal data for volume changes in amygdala subregions with disease progression. Our findings suggest that amygdala subregions are differentially affected by FTD pathology, and have implications for differential diagnosis and monitoring of disease development and progression in FTD.

## Conflicts of interest

The authors declare that they have no conflict of interest.

## Ethical standards

Informed consent was obtained from all participants prior to assessment in accordance with the Declaration of Helsinki. The study was approved by the South Eastern Sydney Local Health District and the ethics committees of the University of New South Wales and the University of Sydney.

### Supplementary Information

Below is the link to the electronic supplementary material.

Supplementary file1 (DOCX 36 KB)

## Data Availability

The data that support the findings of this study are available from the corresponding author, [OP], upon reasonable request.
