# Supplementary material for: Longitudinal volumetric changes in amygdala subregions in frontotemporal dementia
Source: J Neurol. 2024 Jan 24;271(5):2509–20. doi: 10.1007/s00415-023-12172-5 (PMC11055736; doi:10.1007/s00415-023-12172-5)
Supplement: Supplementary file 1 — Supplementary file1 (DOCX 36 KB) [file 415_2023_12172_MOESM1_ESM.docx]

***Supplementary Table 1.*** Amygdala total and subregional volumes at baseline.

|  | bvFTD | SD | PNFA | AD | HC | *F* | *P*-value | Effect size (η²) | Post-hoc (Sidak) |
| --- | --- | --- | --- | --- | --- | --- | --- | --- | --- |
| **Basolateral** |  |  |  |  |  |  |  |  |  |
| Left | 0.79 ± 0.14 | 0.53 ± 0.07 | 0.91 ± 0.12 | 0.82 ± 0.13 | 0.96 ± 0.09 | 41.695 | < .001 | 0.637 | bvFTD < PNFA*, HC**  SD < bvFTD**, PNFA**, AD**, HC**  AD < HC* |
| Right | 0.86 ± 0.15 | 0.77 ± 0.14 | 0.98 ± 0.13 | 0.84 ± 0.12 | 0.99 ± 0.08 | 10.847 | < .001 | 0.314 | bvFTD < PNFA*, HC*  SD < PNFA**, HC**  AD < PNFA*, HC* |
| **Superficial** |  |  |  |  |  |  |  |  |  |
| Left | 0.14 ± 0.03 | 0.10 ± 0.01 | 0.16 ± 0.02 | 0.14 ± 0.02 | 0.17 ± 0.02 | 26.811 | < .001 | 0.530 | bvFTD < HC**  SD < bvFTD**, PNFA**, AD**, HC**  AD < HC* |
| Right | 0.15 ± 0.03 | 0.14 ± 0.03 | 0.17 ± 0.03 | 0.15 ± 0.02 | 0.18 ± 0.02 | 8.742 | < .001 | 0.269 | bvFTD < PNFA*, HC*  SD < PNFA*, HC**  AD < PNFA*, HC* |
| **Centromedial** |  |  |  |  |  |  |  |  |  |
| Left | 0.033 ± 0.009 | 0.023 ± 0.007 | 0.040 ± 0.010 | 0.034 ± 0.007 | 0.044 ± 0.007 | 18.752 | < .001 | 0.441 | bvFTD < HC**  SD < bvFTD*, PNFA**, AD**, HC**  AD < HC* |
| Right | 0.036 ± 0.009 | 0.040 ± 0.012 | 0.047 ± 0.011 | 0.035 ± 0.005 | 0.047 ± 0.008 | 7.881 | < .001 | 0.249 | bvFTD < PNFA*, HC*  AD < PNFA*, HC** |
| **Total** |  |  |  |  |  |  |  |  |  |
| Left | 0.96 ± 0.18 | 0.66 ± 0.08 | 1.10 ± 0.15 | 0.99 ± 0.16 | 1.17 ± 0.11 | 40.249 | < .001 | 0.629 | bvFTD < PNFA*, HC**  SD < bvFTD**, PNFA**, AD**, HC**  AD < HC* |
| Right | 1.04 ± 0.18 | 0.95 ± 0.17 | 1.19 ± 0.17 | 1.02 ± 0.14 | 1.22 ± 0.11 | 10.719 | < .001 | 0.311 | bvFTD < PNFA*, HC*  SD < PNFA**, HC**  AD < PNFA*, HC* |
| Asymmetry index | 4.23 ± 8.55 | 17.94 ± 9.50 | 3.99 ± 5.21 | 1.63 ± 4.91 | 2.01 ± 3.72 | 20.210 | < .001 | 0.460 | bvFTD, PNFA, AD, HC < SD** |

*Note*: Values are means ± standard deviations.

*Abbreviations*: bvFTD, behavioural variant frontotemporal dementia; SD, semantic dementia; PNFA, progressive nonfluent aphasia; AD, Alzheimer’s disease; HC, healthy controls

η² Eta squared

**p* < .05; ***p* < .001

***Supplementary table 2***. Annual percentage reduction of amygdala total and subregional volumes.

|  | bvFTD | SD | PNFA | AD |
| --- | --- | --- | --- | --- |
| Basolateral |  |  |  |  |
| Left | 0.66 (0.58, 0.71) | 1.26 (1.09, 1.47) | 1.23 (1.09, 1.35) | 1.16 (1.02, 1.24) |
| Right | 0.57 (0.50, 0.70) | 4.33 (3.99, 4.73) | 1.65 (1.53, 1.83) | 1.10 (0.99, 1.22) |
| Superficial |  |  |  |  |
| Left | 1.03 (0.95, 1.13) | 0.37 (0.33, 0.43) | 1.42 (1.32, 1.78) | 1.56 (1.21, 1.71) |
| Right | 1.00 (1.15, 1.34) | 3.79 (3.51, 4.13) | 2.29 (2.16, 2.44) | 2.21 (2.05, 2.38) |
| Centromedial |  |  |  |  |
| Left | 3.03 (2.37, 3.45) | 2.78 (2.06, 3.48) | 3.33 (2.88, 3.92) | 4.90 (4.02, 5.35) |
| Right | 2.86 (1.75, 3.23) | 4.63 (4.17, 6.06) | 4.35 (4.00, 5.43) | 2.70 (2.44, 3.03) |
| Total |  |  |  |  |
| Left | 0.76 (0.71, 0.86) | 1.08 (0.94, 1.26) | 1.34 (1.20, 1.45) | 1.31 (1.20, 1.44) |
| Right | 0.71 (0.64, 0.80) | 4.27 (3.94, 4.68) | 1.85 (1.70, 2.00) | 1.31 (1.20, 1.47) |

*Note.* Values are means (95% confidence intervals). Longitudinal atrophy rates were expressed as annual percentage change using the formula: (TIV-corrected most recent volume – TIV-corrected baseline volume)/TIV-corrected baseline volume/time intervals *100%.

*Abbreviations*: bvFTD, behavioural variant frontotemporal dementia; SD, semantic dementia; PNFA, progressive nonfluent aphasia; AD, Alzheimer’s disease; HC, healthy control.
